# A state-of-the-art review on the MicroRNAs roles in hematopoietic stem cell aging and longevity

**DOI:** 10.1186/s12964-023-01117-0

**Published:** 2023-04-24

**Authors:** Geovanny Genaro Reivan Ortiz, Yasaman Mohammadi, Ahmad Nazari, Mehrnaz Ataeinaeini, Parisa Kazemi, Saman Yasamineh, Bashar Zuhair Talib Al-Naqeeb, Haider Kamil Zaidan, Omid Gholizadeh

**Affiliations:** 1 Laboratory of Basic Psychology, Behavioral Analysis and Programmatic Development (PAD-LAB), Catholic University of Cuenca, Cuenca, Ecuador; 2 Faculty of Dentistry, Islamic Azad University, Shiraz Branch, Shiraz, Iran; 3 Tehran University of Medical Sciences, Tehran, Iran; 4 Iranian Hospital, Dubai, UAE; 5 Faculty of Dentistry, Ilam University of Medical Sciences, Ilam, Iran; 6 Stem Cell Research Center at, Tabriz University of Medical Sciences, Tabriz, Iran; 7 Anesthesia Technology Department, Al-Turath University College, Al Mansour, Baghdad, Iraq; 8 Department of Medical Laboratories Techniques, Al-Mustaqbal University College, Hillah, Babylon, Iraq; 9 Research Center for Clinical Virology, Tehran University of Medical Sciences, Tehran, Iran

**Keywords:** MicroRNA, Hematopoietic stem cell, Aging, Anti-aging

## Abstract

Aging is a biological process determined through time-related cellular and functional impairments, leading to a decreased standard of living for the organism. Recently, there has been an unprecedented advance in the aging investigation, especially the detection that the rate of senescence is at least somewhat regulated via evolutionarily preserved genetic pathways and biological processes. Hematopoietic stem cells (HSCs) maintain blood generation over the whole lifetime of an organism. The senescence process influences many of the natural features of HSC, leading to a decline in their capabilities, independently of their microenvironment. New studies show that HSCs are sensitive to age-dependent stress and gradually lose their self-renewal and regeneration potential with senescence. MicroRNAs (miRNAs) are short, non-coding RNAs that post-transcriptionally inhibit translation or stimulate target mRNA cleavage of target transcripts via the sequence-particular connection. MiRNAs control various biological pathways and processes, such as senescence. Several miRNAs are differentially expressed in senescence, producing concern about their use as moderators of the senescence process. MiRNAs play an important role in the control of HSCs and can also modulate processes associated with tissue senescence in specific cell types. In this review, we display the contribution of age-dependent alterations, including DNA damage, epigenetic landscape, metabolism, and extrinsic factors, which affect HSCs function during aging. In addition, we investigate the particular miRNAs regulating HSCs senescence and age-associated diseases.

Video Abstract

**Graphical Abstract:**

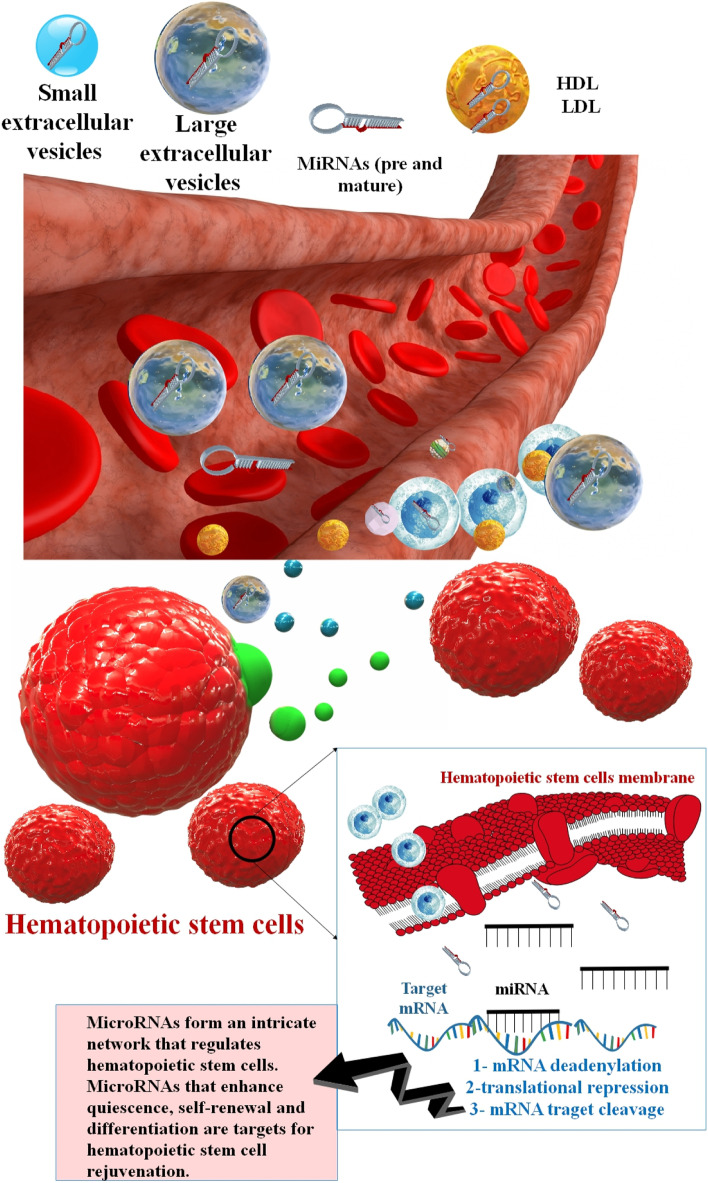

**Supplementary Information:**

The online version contains supplementary material available at 10.1186/s12964-023-01117-0.

## Introduction

Senescence is an unavoidable process. As the older population grows, decreasing aging and potentially age-related disorders require minimizing or controlling senescence. Stem cell therapy has become a promising method for intervening in aging frailty and aging-related disorders [1]. Somatic stem cells include neural stem cells (NSCs), hematopoietic stem cells (HSCs), mesenchymal stem cells (MSCs), hair follicle stem cells (HFSCs), intestinal stem cells (ISCs), and muscle stem cells (MuSCs), which are known as satellite cells of skeletal muscle [2–6].

**Fig. 1:**
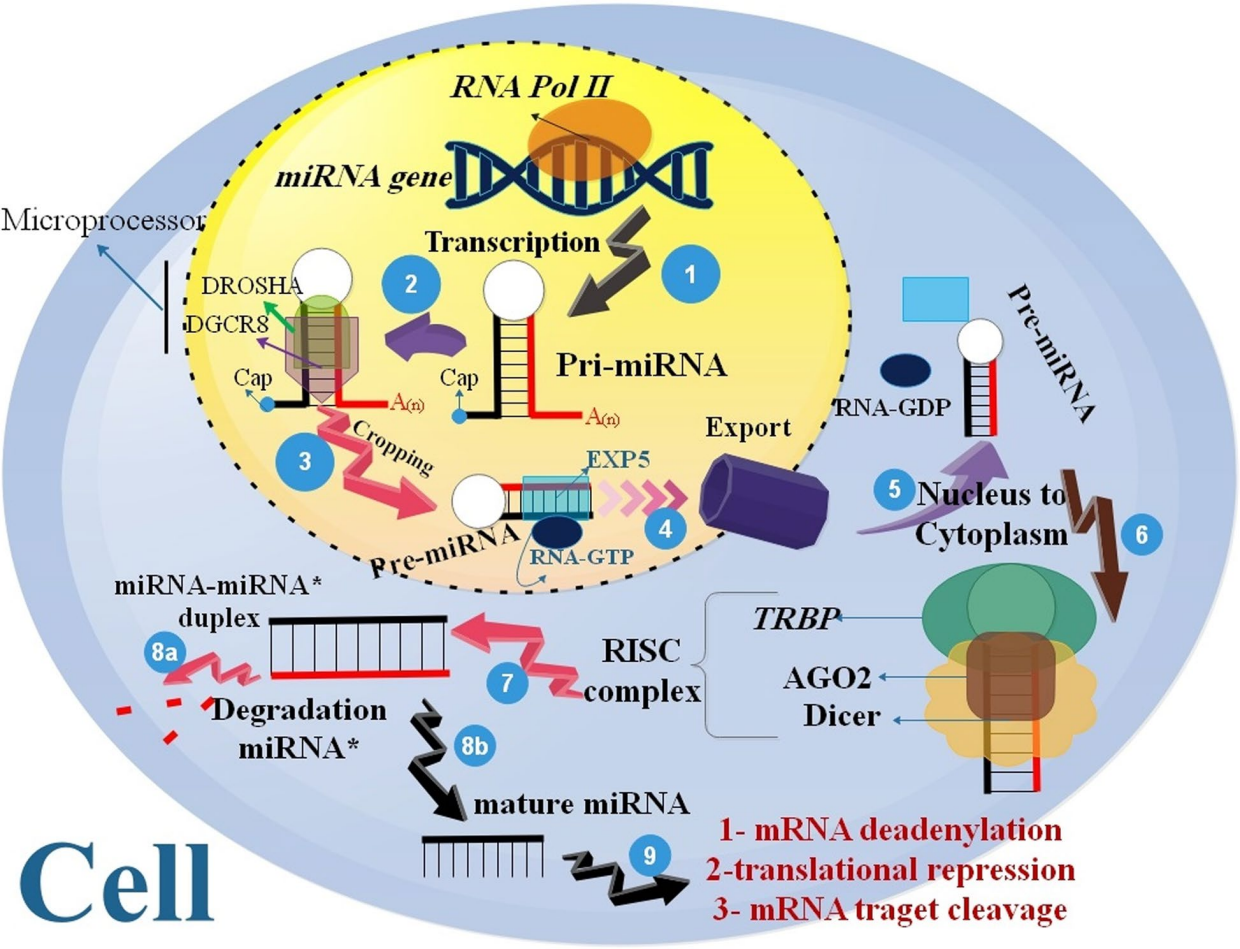
miRNA biogenesis and function in the cell

HSC regulates the hematopoietic system, which produces new blood cells continuously throughout life. Bone marrow (BM) is their primary habitat, although they may also be found in the spleen, thymus, and lymph nodes [7, 8]. They also exist in umbilical cord blood and, in small numbers, in circumferential blood. HSCs play the main role in regulating regular blood cell growth. The BM microenvironment includes a heterogeneous population of stromal cells. They are organized into niches that protect HSCs and other lineage-committed hematopoietic progenitors. Self-renewal or the generation of daughter HSCs, which preserve the HSC pool throughout time, and multilineage differentiation, which generates all the effector cells of the blood and BM, are two of the many characteristics that set HSCs apart from other cells of the hematopoietic system. The stem cell niche generates signals that regulate HSCs self-renewal, quiescence, and differentiation [9–15]. HSCs' in hematopoiesis produce both the myeloid and lymphoid lineages of blood cells, which are in the innate and adaptive immune systems. Myeloid and lymphoid lineages both are included in dendritic cell organization. Myeloid cells include monocytes, macrophages, neutrophils, basophils, eosinophils, erythrocytes, and megakaryocytes, as well as platelets. Lymphoid cells involve T cells, B cells, and natural killer cells (NK) [16–18]. Through a diminishing inclusion that interferes with regular homeostatic tissue maintenance and regeneration response, senescence is likely to play a significant role in the pathophysiology of senescence in many tissues. Companion cells in the BM microenvironment control HSC function [19, 20]. HSCs mediate ongoing blood cell production throughout the organism's lifespan by their protected capacity to self-renew to sustain the stem cell pool and differentiate to give rise to all terminally differentiated blood cells. In adult humans, an estimated one hundred billion new blood cells are produced every day due to the limited lifespan of various effector cells. While the hematopoietic system has various proliferative and regenerative capacities, aging is associated with a general reduction in hematological competence [21, 22]. As with the organization of blood and immune system cells, homeostasis of HSCs occurs when there is a balance between HSC self-renewal and the creation of daughter cells that create specialized lineage-exclusive cells. HSCs are maintained at a constant level throughout an individual's lifespan. To maintain homeostasis, HSCs do not undergo rapid cell division. However, they spend a lot of time in the G0/G1 phase of the cell cycle. Despite the extensive research into HSC maintenance at the molecular level, the processes by which HSCs maintain cellular quiescence remain unknown [23, 24]. Senescence HSCs have been associated with several hematological dysfunctions and pathological alterations, such as skewing the population balance of myeloid cells, lymphoid deficit, decreased immune responses, erythrocytopenia, oligoclonal hematogenesis, myelodysplastic syndrome, and blood cancer [25]. HSCs coexist with osteoblasts (the osteoblast niche), which are regulated by bone morphogenetic protein (BMP). The stromal cell-derived factor 1 (SDF1) adjusts the displacement of HSCs from the blood flow to the BM. The ‌‌BM environments, as well as stromal cells, protect hematopoiesis and produce cytokines such as c-Kit ligand, which stimulates stem cells and progenitors [7]. Wnt signaling is an essential part of the mature stem cells self-renewal and embryonic hematogenesis. The Wnt pathway cascade has various signal transfer contingencies, known as canonical (Wnt/β-catenin) and non-canonical pathways. These two pathways are included in complex operations, including fetal growth, stem cell preservation, and tissue homeostasis. For example, non-canonical wnt5A protein enhanced HSC regrowth in ex vivo conditions. As well as, wnt3a protein enhanced mice HSC self-renewal in vitro. In addition, prostaglandin E2 (PGE2) influences on β-catenin resistance, and also PGE2 persuades canonical Wnt pathway in ex vivo modulation of human cord blood HSC [26]. Notch signaling is necessary for primary HSC growth; however, it is unnecessary for the preservation of mature BM HSCs [27] (Fig. 1).

MicroRNAs (miRNAs) are a group of short non-coding RNA (about 22 nt) that can control the expression of several protein-coding mRNA transcripts by connection to the 3' UTR of target transcripts and inhibiting their translation into the encoded protein or activation of their instability and cleavage of mRNA [28]. Numerous reports have shown that miRNA functions as unique expression templates in the hematopoietic system, with specific miRNAs having the ability to affect the maturation of distinct blood cell lineages. Different miRNAs, including miR-22, miR-29a, miR-125a, miR-126, and the miR-132/122 cluster, have been demonstrated to play crucial functions in HSC biology [29]. By identifying and confirming mRNA targets, miRNA regulatory networks in senescence HSCs and tissues may provide opportunities for HSCs in vitro and in vivo [30].

In this review, we display the contribution of age-dependent alterations, including DNA damage, epigenetic landscape, metabolism, and extrinsic factors that affect HSCs function during aging. In addition, we discuss the roles of the particular miRNAs regulating HSCs senescence and age-associated diseases.

## MiRNAs function in stem cell aging

**Fig. 2:**
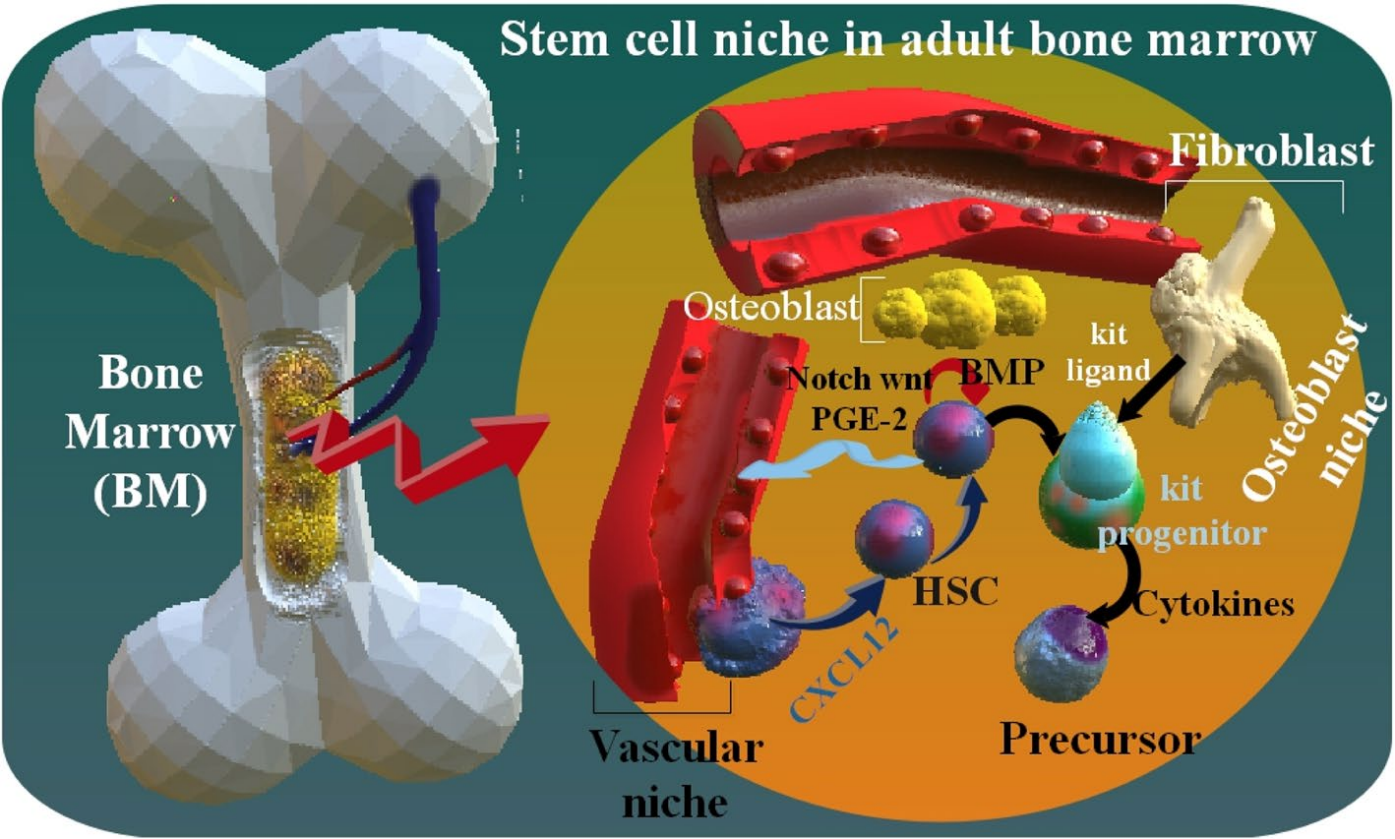
Maturated bone marrow (BM) stem cell niche. In the osteoblast niche (by using Notch, Wnt, and PGE-2 pathways), HSCs exist near the osteoblast, which is regulated by bone morphogenetic protein (BMP). In addition, HSCs are as well as exist near the blood vessels in the vascular niche. The stromal cell-derived factor 1 (SDF1), as well as recognized as C-X-C motif chemokine 12 (CXCL12), controlled the immigration of HSCs in the blood flow to the BM. In vivo, the osteoblast and vascular niches may be close to one another. The BM environment also includes stromal cells, which protect hematopoiesis, such as the generation of cytokines, including c-Kit ligand, which was induced by stem cells and progenitors

MiRNA genes are transcribed through RNA polymerase II (pol II) and may be synthesized either from their genes or from a segment of sequences in protein-coding genes. MiRNAs are derived from longer ds-RNAs named pri-miRNAs, which may be produced from intergenic regions, exonic or intronic sequences, or as polycistronic transcripts (including many hairpin structures in a single RNA transcript) [31, 32]. The pri-miRNAs are cleaved into hairpin-formed premature miRNA (recognized as pre-miRNA) via the catalytic RNase III domain of Drosha. Pre-miRNA hairpins are transferred from the nucleus to the cytoplasm through a RanGTP/exportin 5-related system. Dicer (RNase III) converts the pre-miRNA hairpin into the mature 22 nt double-stranded miRNA*/miRNA duplex in the cytoplasm [33–36]. Multiple proteins were used to assemble an RNA induced silencing complex (RISC) with a single strand deleted and a single strand protected as a guide strand, which can connect to target mRNAs as a supplement, suppressing translation, mRNA instability, and/or mRNA split for post-transcriptional regulation of protein synthesis [37]. The methods miRNA to suppress of target mRNAs or to regulate the protein-coding genes, including suppression of elongation (mRNAs inhibition), suppression of translation (Cap and 60S Joining suppression), ribosome drop-off (premature termination), Co-translational protein destruction [34, 38–41] (Fig. 2).miRNAs are implicated in several biological processes, including developmental timing, differentiation, apoptosis, stem cell growth and development, immune reaction, aging, and cancer [42]. In addition, miRNAs and aging presumably play an intertwined function in driving these pathologic conditions. New research has shown that miRNAs play a role in the aging of stem cells. miRNAs are a shape of epigenetic control that changes gene expression without altering genetic code [43]. One of the first sets of miRNAs proposed for the stem cells regulation was the let-7 family. *Caenorhabditis elegans* was used to describe this conserved family of miRNAs throughout evolution. Similarities between let-7 in *C. elegans* and the mouse are observed by Nishino and coworkers, are intriguing. Hypodermal stem cells (seam cells) of *C. elegans* are strongly stimulated in let-7 near the end of their differentiation process, and impairment of let-7 activity results in the ongoing proliferation of these cells [44]. Furthermore, miRNAs are epigenetic modulators of gene expression that inhibit or repress the translation of specific mRNAs. Many studies have used miRNAs to target oncogenes, tumor suppressors, and differentiation markers, all of which need to be suppressed to maintain stem cell self-renewal [45]. Blood transfusions from young mice into old mice have shown improvements in cognitive performance and synaptic plasticity, as well as restoring the regenerative capacity of skeletal muscle stem cells, as part of several studies looking into parabiosis as a means of rejuvenating older animals. Several studies have shown the presence of miRNAs in blood plasma and serum. In addition, as age progressed, changes occurred in the expression of miRNAs and the mRNAs they target in peripheral blood mononuclear cells (PBMC). The function of several miRNAs in degenerative disorders associated with aging has been confirmed. The potential use of miRNAs as therapeutic targets has been the subject of recent research, and new studies elucidating their precise function are now being published [46].

## HSCs Aging

Mechanisms that cause cellular senescence might be intrinsic alterations such as telomere friction, proteostasis changes, epigenetic viewpoint changes, DNA damage, mutational load, and mitochondrial failure. Foreign modifications may also vary from small niche-macroenvironmental changes to systemic level changes to larger-level environmental insults such as irradiation, pathogen, and reactive oxygen exposure [47–53] (Table 1).

**Table 1:** Alterations in stem cell characteristics with age [54]

**Stem cells**	**Self-renewal in senescence tissues**^a^	**Proliferative activity**	**Differentiation capability**	**Regeneration and repair**
HSCs	About × 2–6	diminished	Increased myeloid cell production	Immune suppression, reduced engraftment potential
NSCs	About ÷ 2	diminished	Maintained in vitro	-
MuSCs	About ÷ 2	diminished	Increased fibrosis after injury	Myofibril regeneration and reduced engraftment potential
ISCs	=	diminished	Increased secretory lineage cells	UV exposure reduces generation; response delayed
HFSCs	=	diminished	=	The hair cycle stops, and wounds take longer to heal

^a^Increased ( ×)/ Decreased ( ÷)/Maintained at equivalent levels ( =)

**Fig. 3:**
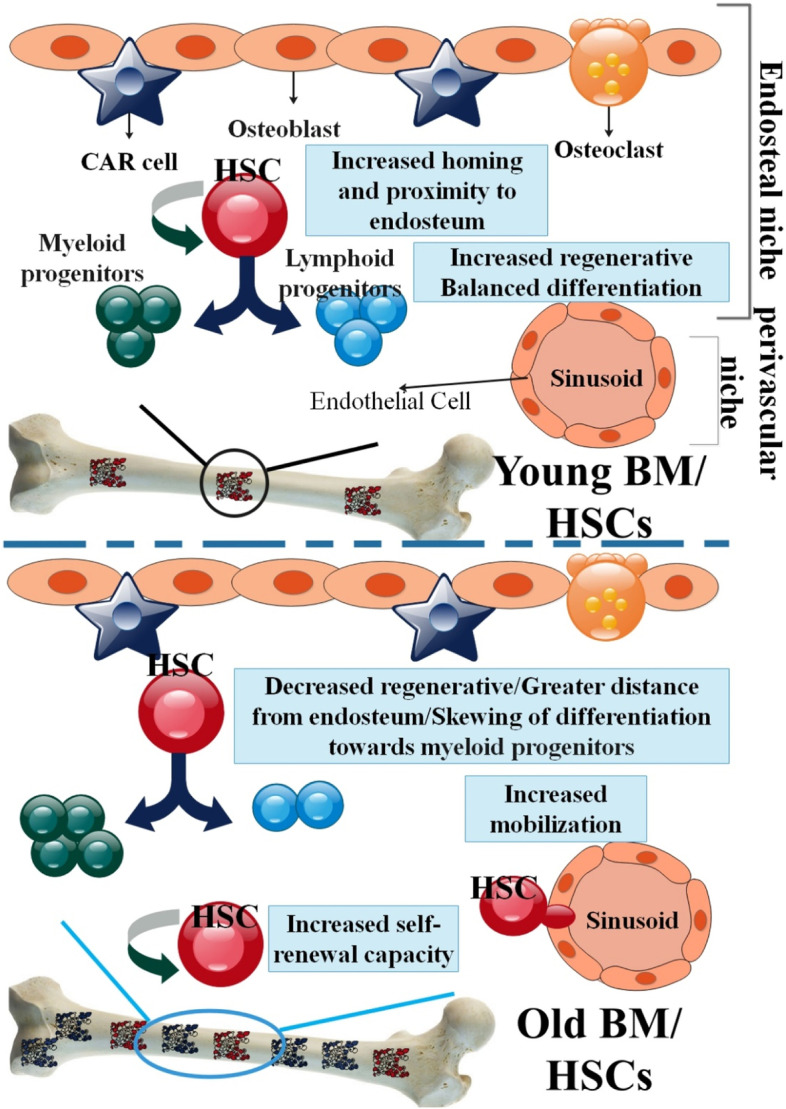
The schematic comparison of aged and young HSCs function in BM. While the total number of cells with regenerative potential in the BM of elderly adults increases, the extent to which specific old cells can still chip away at blood cell production becomes highly variable. Young HSCs are home to the BM and centralize near endosteum. They have great self-renewal and regenerative potential and a moderate differentiation ability towards lymphoid and myeloid progenitor cells. The location of elderly HSCs in the BM is distinguished from that of young HSCs; elderly HSCs centralize away from the endosteal stem cell niche following their transplantation

Similar processes occur during the maturation of blood cells in both mice and humans. Therefore, it is likely that the exact mechanisms that induce stem cell senescence in mice also do so in humans [55]. The contribution of the systemic environment to the regeneration of aging tissues and stem cells was recently shown in groundbreaking experimental studies. The cognitive performance and physical stamina of geriatric mice models have been shown to improve after receiving transfusions of young blood. For instance, injecting young blood into the body led to an increase in growth differentiation factor 11 (GDF11) levels, a restoration of muscle structure and function, and improved strength and stability exercise in a mouse model of aging [56–58]. HSCs are increased in aged humans or mice. As a result, the number of HSCs population is determined by its surface markers. By applying clonal assays, recent studies have shown both quantitative and qualitative alterations of HSCs in senescence period [15, 59]. Human observations shown that the number of immunophenotypically determined HSCs or progenitor cells from healthy men enhances with aging and causes a reduction in their self-renewal ability and quiescence state [60, 61]. In an investigation, clonal assays and single-cell RNA sequencing were used to examine variations in proliferation and self-renewal capabilities. They reported that aged HSCs can directly influence the populations of innate and acquired immune cells. Also, the unique characteristic of senescence HSCs is their disproportionate focus on the myeloid lineage during differentiation at the expense of the lymphoid lineage [15, 62]. In aged tissues or organs, the equilibrium between HSC self-renewal, action, and durability is strongly altered. Young HSCs produce a balanced population of myeloid and lymphoid progenitor cells. However, aging causes an increase in the differentiation of HSCs to myeloid progenitor cells, resulting a decrease in the formation of B and T cells. The changed combination of the hematopoiesis can be accountable to the immune senescence phenotype Known in aged persons. Senescence HSCs are characterized by improved self-renewal, diminished long-term regeneration capacity, myeloid-biased differentiation, and niche localization variance. Consequently, older mice demonstrate a repositioning of phenotypically defined HSCs with a poor capacity to home to the BM niche [63, 64] (Fig. 3). Several molecular and cellular pathways contribute to the decline in HSC function that occurs with aging. A variety of variables and processes, including cell cycle-dependent genes and epigenetic modifications, have been examined in HSC senescence as a means of assisting HSCs in adapting to aging process. For example, a change in *p53* activity affecting HSCs numbers, proliferation capability, and hematopoiesis in aged organisms, support a model in which aging is caused by a reduction in tissue stem cell regenerative function [63, 65–67]. Loss of polarity in aged HSCs coincides with the expression of the RhoGTPase Cdc42, which is directly associated with HSC senescence. Functionally rejuvenating old HSCs by blocking Cdc42 activity with a drug, increases the proportion of polarized cells in an aged HSC population and returns the amount and spatial repartition of histone H4 lysine 16 acetylation to that of young HSCs. In addition, a pharmaceutical target for reducing stem cell aging and elucidating a molecular function for Cdc42 activity in HSC biology and epigenetic control [68]. In addition, a meta-analysis employing mice HSCs uncovered a link between HSC decline and epigenetic modifications as people age [69].

### Metabolism of HSCs aging

Metabolic processes are an organism's chemical reactions that keep it alive. Quiescence to reduce stress damage, proliferation, and self-renewal to maintain progenitor pools, and lineage specification for tissue regeneration represent metabolically distinct stem cell appreciations of different energy sources. The primary purposes of metabolism are included: proteins, fatty acids, nucleic acids, and some carbohydrates, as well as the removal of nitrogenous wastes. Hydrocarbons and energy in the form of ATP, and dwindling cofactors from catabolic productions are substrates for the anabolic production of non-renewable macromolecules. Metabolic circulation supplies energy and activates master genetic programs that control cells behaviour [70–72]. To prevent cellular damage from reactive oxygen species (ROS) and maintain their tissue-renewing capacities throughout life, quiescent somatic stem cells maintain a slow metabolic rate [73]. Recent research shows that variations in stem cell populations are nutrient-affiliated. Nutrient sensing signaling, for example, the balance between quiescence and proliferation in aging stem cells, is regulated by a several pathways, including the mammalian target of rapamycin (mTOR), Protein Kinase B (Akt), and AMP-activated protein kinase (AMPK) [74]. Many animals benefit from caloric restriction by extending their lives and slowing the onset of age-related diseases, thus, researchers have been looking into the mechanism by which this occurs in stem cells [75, 76]. Cellular activity and proliferation are boosted by caloric restriction via mTOR, IGF, and MAPK signaling. Somatic stem cells proliferation are restricted by ROS in a hypoxic niche, suggesting that environmental factors may play a role in stem cell aging. The reactivation of hypoxia-inducible factors is directly linked to the reactivation of stem cell quiescence, proliferation, and oxidative metabolism [77–80]. Sirtuins play an essential role in the cellular reaction, environmental stress, promoting DNA repair, telomere consistency, cell cycle arrest, cellular senescence, and apoptosis. The function of sirtuins in natural longevity is considered [81, 82]. Sirtuins 2, 3, and 7 all decrease with aging in HSCs, and maintaining their expression in old HSCs can reduce mitochondrial stress and enhance HSC function [83]. Sirtuins 7 inactivation led to decreased quiescence, enhanced mitochondrial protein folding stress (PFS(mt)), and compromised regenerative capability of HSCs. Sirtuins 7 expression was reduced in old HSCs, and Sirtuins 7 upregulation ameliorated the regenerative capacity of aged HSCs. Mitochondrial unfolded protein response (UPR(mt)) is interceded through the interaction of SIRT7 and nuclear respiratory factor 1(NRF1) and is associated with cellular energy metabolism and proliferation. These data implicate dysregulation of a UPR(mt)-interceded metabolic checkpoint as a reversible contributing agent for HSC senescence [84].

Numerous molecular and cellular essential pathways have been identified as factors in the decline of HSC function with age. Mechanistically, however, it may be possible and beneficial to communicate these multiple aging pathways separately, leading one to conclude that they are, in fact, highly related and connected. While it's implausible to reversed any of the cellularly fundamental causes of aging, number of them have the potential to be intervened on and thus could be a target for pharmacological study [63]. Downstream transcription factors activated in situations of low IIS activity, Foxo proteins (FOX (Forkhead box)), promote quiescence, long-range preservation, and the inclusion of a variety of somatic stem cell populations in flies and mice, all of which are necessary for tissue repair and regeneration [85, 86]. The conservation of this Foxo function in mammalian HSCs highlights its importance in controlling stem cell quiescence. To some extent, Foxo's capacity to control antioxidant gene expression mediates this effect since HSCs with mutant Foxo show elevated levels of reactive ROS. Furthermore, Foxo loss-of-function phenotypes may be rescued by treatment with the free radical scavenger N-acetyl cysteine (NAC) [87–89].

### DNA damage of aged HSCs

Stem cell lineages in various organs and tissues become more susceptible to DNA damage as we age [90, 91]. Another major cause of stem cell senescence is dysfunction in DNA damage repair. DNA damage, in turn, causes a particular DNA damage response (DDR), which includes the following occurrences, a) triggering of any each kinase (ATM, ATR, DNA-PK), b) phosphorylation of adaptor protein 53BP1, and c) creation of the discrete foci, comprising phosphorylated histone H2A.X and p53BP1. In addition, DDR triggering results in cell cycle arrest via triggering of p53/p21 and/or p16/pRb pathways. The hydroxyl radical, the most biologically energetic free radical, is the dominant reactive oxygen species (ROS) that target DNA. It is generally accepted that oxidative stress and ROS ultimately lead to DNA damage, whereby inadequate cellular restoration mechanisms may chip into premature aging and apoptosis. In the aged cells, increased ROS can lead to direct DNA damage and continuous DDR triggering, thus forming a feedback loop [92–94]. Increased DNA damage may lead to alterations in gene function due to mutations or chromosomal rearrangements. Although somatic stem cells are given a leg up in the cell cycle and metabolism, these advantages may be lost with age or function due to the robust activation of the DNA damage response and the subsequent activation of tumor suppressor genes [95–98]. A cell-intrinsic factor that induces HSC senescence is discussed DNA damage. HSCs are accountable for preserving tissue homeostasis during a lifetime. Therefore, it is crucial for HSCs to maintain their genomic integrity to decrease the danger of BM failure or transformation. The DNA damage theory of stem cell aging explains aging-related alterations in the DNA repair system in HSCs with alterations in cell division control, arising from enhanced DNA damage with age, which may lead to increased DNA mutations. Then, with increasing age, the function of HSCs decreases [99]. Studies in mice and human patients with mutations in genes-producing proteins involved in DNA repair provide essential insights into the early senescence of stem cells. As DNA damage accumulates with age, the functional capacity of HSCs decline, a process known as physiological senescence [95–98]. In addition to a loss of proliferative capacity, decreased self-renewal, and functional exhaustion, HSCs from mice deficient in DNA damage maintenance also showed signs of cellular exhaustion. For instance, γ-H2AX foci and other markers of extensive DNA damage accumulate in elderly HSC over time [97, 100]. It is still unclear whether or not genetic damage is the actual cause of HSCs' aging. In general, it is difficult to comprehend how the buildup of DNA damage may directly lead to stem cell dysfunctioning if HSCs are truly primarily quiescent and divide relatively seldom throughout a mouse's lifespan. Myeloid-biased HSCs have been demonstrated to be included in the quiescent state, and it is possible that the cells immediately downstream of these HSCs are targeted for DNA damage accumulation [101, 102]. In addition to random DNA damage, it has been shown that DNA mutations at specific loci are linked to the onset of clonal hematopoiesis in otherwise healthy elderlies. Telomere abrasion causes a different kind of DNA damage. The failure to maintain telomere length is linked with challenging HSC dysfunction since the role of telomere shortening in the functional decline of HSC is only apparent in humans and mice with long telomeres. Although HSC telomere length may be increased by forced overexpression of telomerase, doing so does not restore functional damage in mice [103–106]. Furthermore, external agents, inherent changes that are not mutations in DNA, might finally contribute to HSC senescence. Researchers showed that HSCs alter their polarity on senescence in both the cytoplasm and the nucleus. Therefore, changes in overall cell structure may also contribute to HSC senescence. Alterations in the three-dimensional arrangement of epigenetic marks and structural proteins might affect the cell cycle in a way that decreases capability in daughter stem cells, for instance, helping in the natural senescence of HSCs. Generally, several mechanisms might contribute to the senescence of HSCs and ultimately relate to the interplay between internal and external cell agents [99].

### The epigenetic basis of HSC aging

**Fig. 4:**
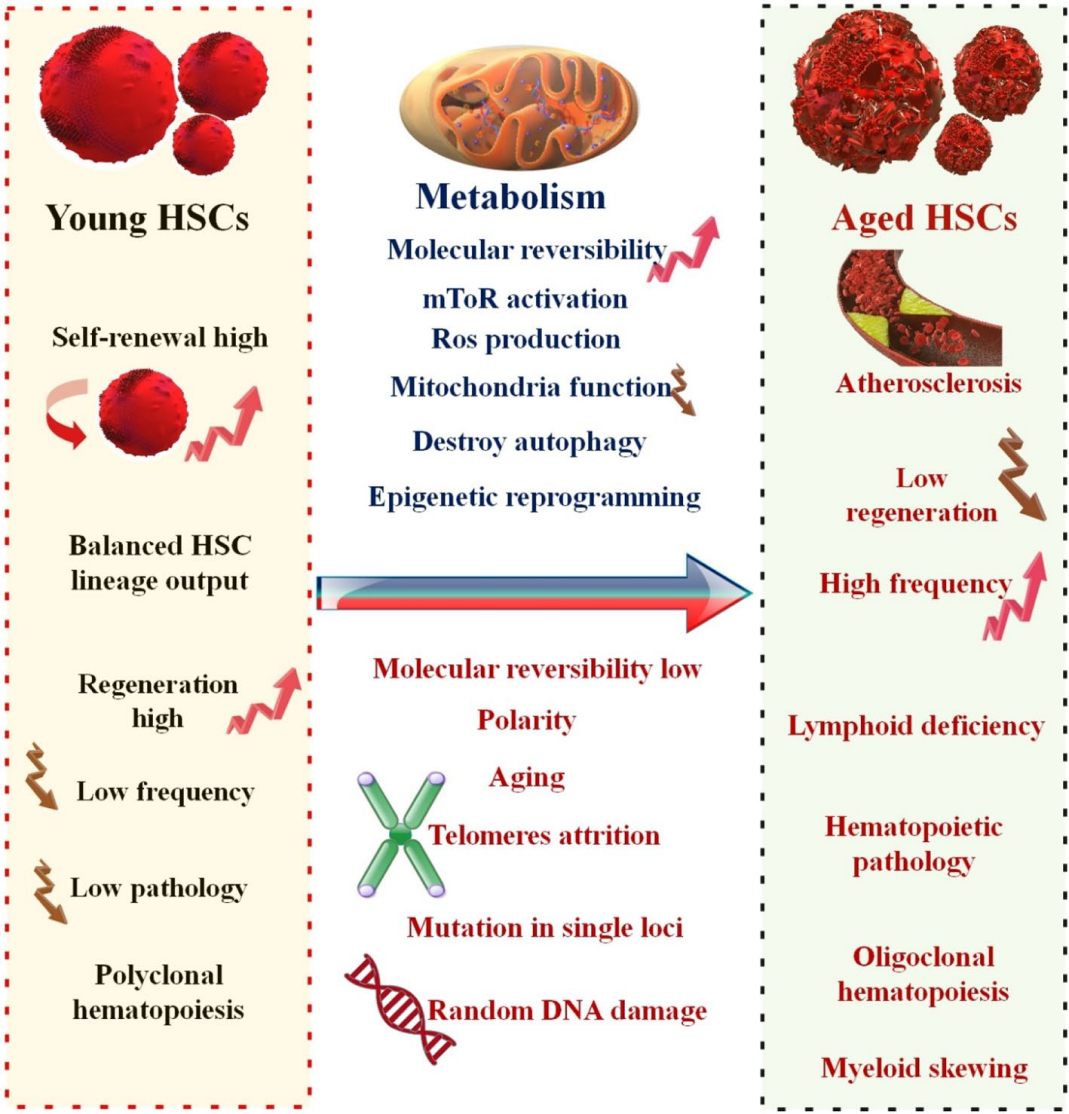
Essential pathways that aid senescence in HSCs. Although it may be challenging to restore some molecular events, others may be amenable to pharmacological interpositions and therefore be exploitable in the context of HSC rejuvenation

Epigenetics examines how changes in gene expression may be passed down from generation to generation to affect cellular phenotype independent of DNA sequence. In a broader sense, the word refers to the mechanism of genomic control that is not based on the sequence of nucleotides [107–110]. There are several kinds of epigenetic information encoded within our epigenome, which it is not limited to the existence or lack of histones on any specific DNA sequence, such as DNA methylation, chromatin remodeling, posttranslational modifications of the histone proteins, structural and functional variants of histones, and transcription of non-coding RNAs (ncRNAs). Different studies show that epigenetic regulators are essentially needed for the preservation of tissue-particular stem cells and epigenetic marks are changed during physiological aging in stem cells [111, 112]. Similar to cells terminal differentiation to skin cells, liver cells, brain cells, etc., epigenetic alterations may show up in a wide variety of ways. On the other hand, epigenetic alteration may have much more dire consequences, including the cancer development. At present, epigenetic modification is evaluated on its ability to start and maintain at least three systems: DNA methylation, histone modification, and non-coding RNA (ncRNA)-associated gene silencing [107–110]. Activation and repression of genes, which play regulatory roles in transcription initiation and elongation, include various histone modifications. Moreover, the age-related altered expression of chromatin-modifying enzymes may generate epigenetic alterations in aged stem cells. Changes in histone modifications and chromatin remodeling proteins have been extensively studied for aged stem cells. For example, the transcriptional repressors of the polycomb group restrict the aging process by marking the INK4a locus with the repressive histone marker H3K27me3 [113, 114]. DNA methyltransferase 1 (DNMT1) is a protein-coding gene with a crucial role in HSCs and when the gene is genetically inactivated, its deficiency result in the near-total elimination of HSCs in living organisms. Additionally, HSCs from mice with reduced Dnmt1 activity become restricted to myeloerythroid differentiation as a result of the devastating silencing of essential lineage determinative genes such as Gata1, Id2, and CEBP/, as well as a dysfunction to prime master lymphoid regulators like Ebf1, Pax5, and Il7r20 [115–117]. Changes in the DNA methylome are associated with senescence in HSCs. The hypermethylation phenotype shared by aging HSCs and senescence post-mitotic somatic cells is characterized by a gradual increase in all DNA methylation levels. The mechanism for HSC hypermethylation in aging has not been fully explained. Collectively, DNA methyltransferase enzyme-encoding genes are repressed in aged HSCs, in contrast to their expression in youthful HSCs. However, this does not explain why and how distinct isoforms of Dnmt3a and Dnmt3b are expressed and functional [69, 118]. By directly inhibiting DUSP1 with repressive histone marks, BMI1 increased COX-2/PGE2 production, which is crucial for immune preventive properties. It has been shown that BMI1 also helps human HSCs maintain their quiescent state for longer, allowing for more self-renewal [119]. The histone deacetylase Sirt1 is essential for stem cell homeostasis and has been related to the loss of stem cell function in aging and illness. Sirt1, a chromatin modulator, maintains HSC homeostasis by altering Hoxa expression via epigenetic regulation. After Sirt1 deletion, an increase in H4K16 acetylation and a reduction in H3K27 trimethylation led to an up-regulation of Hoxa9. H3K27me3, an inhibitory marker, also increased in both HSCs with age. Age-related loss of lymphoid differentiation capability in HSCs was mirrored by a raised pattern of H3K27me3 [118, 120, 121] (Fig. 4).

## miRNAs in HSCs aging

During each stage of differentiation, a unique miRNA signature is produced by HSCs. By regulating the expression of the master pluripotency genes and early organogenesis, miRNAs have been shown to play a role in maintaining "stemness" and priming differentiation. MiRNAs constitute an additional regulatory mechanism in HSCs, influencing transcription patterns and transcript consistency. There is evidence that miRNAs can direct primary somatic cells back to a pluripotent state [122]. Numerous unique miRNAs have been identified with a specific impact on the behavior of stem cells when their expression is disrupted in the human hematogenesis pathway. Eliminating this gene results in increased differentiation, suggesting that miR-23a suppresses differentiation, in contrast to the pro-differentiation effects of other miRNAs such as miR-181, miR-223, and miR-142. Reducing the number of HSCs and HSPCs is the net effect of eliminating miR-23a and the closely related miR-23b. The intricacy of miRNA networks, regulating HSCs is demonstrated by these examples and the observation of miRNAs with several mRNA targets [123, 124]. Targeting genes involved in DNA damage, epigenetic modifications, and metabolism, miRNAs control HSC aging. Here, we'll consider the roles of exogenously introduced miRNAs and the pathways they're involved in as HSCs age, along with the direct targets of those miRNAs [125].

### The miR-212/132 cluster (Mirc19)

Researchers showed that the miRNA-212/132 cluster is increased in HSCs and is upregulated in aging. The miRNA-132 and miRNA-212 overexpression and elimination of these miRNAs result in inappropriate hematogenesis with increasing age. Mice with miR-132 overexpressed in their BM had rapid HSC cycling and depletion. Mice, in whom this cluster of miRNAs had been genetically eliminated, had HSCs with altered cycle, function, and survival in response to growth factor deficiency. In this study, researchers demonstrated that miR-132 targeted the transcription agent FOXO3, an established senescence-related gene, to exert its effect on senescence HSCs. Furthermore, by regulating FOXO3 expression, these miRNAs help maintain a preserving balance in HSCs' production [126].

### miR-125b

miR-125b, which regulates HSC survival, is highly expressed in the early compartment and is regulated by DNA and histone methylation in tumor settings. miR-125b expression rates are lowered in HSC populations with aging. A higher frequency of the CD150low "lineage balanced" and CD150neg lymphoid-biased HSC subsets is seen when miR-125b expression is elevated, suggesting that miR-125b confers a more stress resistant, anti-apoptotic scenario to the HSCs, influencing the composition of the HSC compartment. It is interesting to note that the frequency of these HSC subsets is reduced in aging animals, suggesting that the miR-125b deregulation is involved in the variations of the CD150 compartments frequency [127–129].

### MiR-33

MiR-33 is downregulated in HSCs and strongly expressed in MPPs in super-p53 (sp53) animals with an extra copy of the p53 gene. After transplantation, miR-33 transduced sp53 HSC shows extraordinary regeneration capabilities but drastically reduces recipient survival. In addition, high levels of miR-33 inhibit tumor-derived cell lines' apoptotic response, cause murine embryonic fibroblasts (MEFs) to undergo a neoplastic transformation, and promote MEFs' anchorage-independent proliferation. Downregulation of p53 by miR-33 is associated with its binding to two conserved domains in p53's 3′UTR. To prevent and treat hematological diseases, understanding the role of miR-33 in controlling HSC self-renewal through p53 is crucial [130].

## miRNAs function in HSCs age-related diseases

Myelodysplasia, chronic myelogenous leukemia (CML), polycythemia vera, and leukemia are all clonal hematopoietic diseases that are more common in the elderly and may be caused by the genetic and epigenetic abnormalities that become more common in HSC clones as we age. Some researchers believe that changes in the BM microenvironment that occur with age have a role in the selection of senescence human HSC clones [131]. For instance, when comparing BM-HSPCs from elderly trauma patients to those from younger patients, the latter shows a more muted mRNA/miR reactivity to trauma. Senescence may be the main driver of post-traumatic BM-HSPC transcriptome and specific epigenetic changes, independent of injury severity and blood transfusion need. The reason of poor hematopoiesis response to trauma in older individuals may be explained by the regulation of crucial miRs and genes associated witth HSPC synthesis, and differentiation, leading to the next immunological dyscrasia. Even though HSPC immunomodulation is doable, it's possible that older adults will not respond well to conventional cytokines and growth factors. Long-term effects on the elderly might be improved with epigenetic modification to preserve HSPCs for use in personalized therapy [132]. Age-related changes to the hematogenesis mechanism include heightened inflammation, impaired HSC function, and an increased risk of myeloid malignancy. Age-related changes in HSC role and myeloid malignancy have been linked to inflammation in the elderly (also known as "inflammaging") [133].

### miR-146a

Researchers found that miR-146a deficiency contributed to age-related inflammation in individuals with acute myeloid leukemia (AML). Loss of miR-146a in young miR-146a-null mice enhanced senescence of HSCs and inflammation, and senescence-related AML developed earlier than in wild-type animals. An undeveloped subset of resting HSCs was eliminated after miR-146a inhibition. DNA methylation and transcriptome profiling implicated NF-κB, IL-6, and TNF as potential drivers of HSC dysfunction. This resulted in an inflammatory signaling relay leading to increased IL-6 and TNF release from mature miR-146a myeloid and lymphoid cells. Single-cell measurements of miR-146a HSC involvement and subpopulation creation were restored and when inflammation was reduced by targeting IL-6 or TNF, the incidence of hematological malignancy has reduced miR-146a in mice. Loss of miR-146a alters HSC function through cell-extrinsic inflammatory signals and greater cell-intrinsic sensitivity to inflammation, as shown by miR-146a/ HSCs' heightened sensitivity to IL6 induction. Consequently, HSC inflammation contributes to the formation of AML through cell-extrinsic and -intrinsic pathways regulated by the miR-146a loss [133].

The miR-146a has a crucial role in dampening the inflammatory response. The miR-146a depletion leads to fatigue of HSCs and the development of hematological tumors, reduction in the number and quality of HSCs, and an increase in myeloproliferative neoplasms. The internal problem with miR-146a-defective HSCs, and the extrinsic efficiency of lymphocytes and non-hematopoietic cells contribute to the cellular environment's insufficiency. This contains the miR-146a, the signaling protein TRAF6, the transcription factor NF-κB, and the IL-6 along a molecular axis. Using a mouse model of chronic inflammation, researchers found miR-146a to be a crucial regulator of HSC homeostasis and established a mechanical connection between chronic inflammation, BM failure, and the development of myeloproliferative neoplasms. Myelodysplastic syndromes (MDS) are a hematological malignancy of older persons (median age 70 years) that always exhibited reduced expression of miR-146a, making miR-146a-defective mice an excellent model to investigate the etiology of MDS. It concludes by suggesting that chronic inflammation could be to blame for the age-related decline in HSC activity [134].

Distinct types of hematologic malignancy may be identified by the abnormal growth of lymphocytes, which are known as lymphoproliferative disorders. Transplantation of autologous HSCs is a crucial component of treatment for lymphoproliferative conditions. Existing miRNAs in the hematopoietic niche that target cytokines and signaling pathways may have a significant regulatory role in the mobilization of HSC. Furthermore, miRNAs may influence CD34 + cell mobilization efficiency. Next to the first apheresis, a negative relationship was detected among hsa-miR-146a-5p and the quantity of total CD34 + cells. Compared poor mobilizers, excellent mobilizers had a lower hsa-miR-146a-5p rate on the day of the first apheresis, as determined by GITMO criteria. Potentially boosting HSC mobilization efficiency, Hsa-miR-146a-5p [135].

### miR-126

miR-126 has been determined as an essential modulator of HSCs. Reduced levels of miR-126 caused an increase in HSC cycling, which led to a dramatic increase in the HSC compartment and a corresponding reduction in lymphoid capacity. This functional stem effectiveness is also at odds with AML stem cells due to miR-126's control of normal HSC cycling. In AML stem cells, miR-126 protects quiescence and promotes antineoplastic resistance by targeting the PI3K/AKT/mTOR signaling pathway, as shown by a combination of transcriptome and proteome analysis. These characteristics, except retained complete reconstitution capacity, are again indicative of HSC senescence: development of the HSCs and reduced lymphoid output, and miR-126 is linked as a significant mediator of HSC senescence [128, 136].

## miRNA-based interventions in senescence HSCs

Incorrect quiescence, self-renewal, and differentiation are seen in aged HSCs. As miRNAs can regulate these processes, they may restore homeostasis to a more 'youthful' state. As a result, miR intermediacy presents a promising strategy for revitalizing HSCs. The mTOR inhibitor rapamycin and calorie restriction are two examples of therapies shown to delay senescence. In the latter case, no proof exists that HSCs can be kept young via nutritional therapies. However, rapamycin may have beneficial effects on HSCs that have reached senescence. The serine/threonine protein kinase mTOR, which regulates cell growth, metabolism, and autophagy, is inhibited by rapamycin. Genes in the mTOR pathway are also targeted by miR-21, miR-22, miR-99, miR-125a/b, and miR-155 [123, 137] (Table 2).

**Table 2:** HSCs aging-related miRNAs and their miRNA target(s)

MicroRNA	mRNA Targets	Description	References
miR-212/132 cluster	FOXO3	miR-132 utilized its efficacy on senescence HSCs by targeting the transcription agent FOXO3, a recognized aging-related gene. In addition, these miRNAs have a function in preserving balanced HSCs output	[126]
miR-125b	HOXA1	Overexpression of miR-125b alters the HSC compartment composition by providing HSCs with a more stress-resistant and anti-apoptotic environment, resulting in an increase frequency of the CD150low "lineage balanced" and CD150neg lymphoid-biased HSC subsets	[127–129]
miR-33	p53	Defining the function of miR-33 in regulating the HSC self-renewal via p53 may result in the inhibition and therapy of hematopoietic disorders	[130]
miR-146a	TRAF6	Therefore, loss of miR-146a controls cell-extrinsic and -intrinsic pathways associating HSC inflammation to the development of AML	[133]
miR-139 − 5p	BRG1	miR-139-5p is a crucial modulator of cellular proliferation in primary hematopoiesis and is a strong antileukemic molecule	[138]
miR-126	CDK3	miR-126 targets the PI3K/AKT/mTOR signaling pathway, protecting AML stem cell quiescence and promoting antineoplastic resistance	[128, 136]
miR-193b	c-KIT	Ectopic miR-193b expression limits long-time repopulating HSC development and blood regeneration. miR-193b-defective HSCs and pHSCs show enhanced basic and cytokine-stimulated STAT5 and AKT signaling. This STAT5-stimulated miRNA provides negative feedback for extreme signaling to limit unregulated HSC increase	[139]
miR-382 − 5p	MXD1	miR-382-5p overexpression in CD34 + HSCs/pHSCs results in a remarkable reduction of megakaryocyte precursors coupled to augment granulocyte ones	[140]
miR-155	CXCL12	miR-155 enhances G-CSF-stimulated mobilization of murine HSCs and pHSCs through the propagation of CXCL12 signaling	[141]
miR-143/145	TGFβ	miR-143/145 plays a cell context-related function in HSPC action via control of TGFβ/DAB2 triggering, and lack of these miRNAs generates a preleukemic condition	[142]

## Conclusion

During senescence, HSCs undergo an ongoing disorder of function accompanied by a decreased regenerative capability. Understanding the many biochemical processes driving the malfunctioning of senescence HSCs is a critical focus of biomedical research. The average age of the general population is increasing as new health care advances. If molecular therapies that regenerate senescence HSCs are discovered, it might reduce the burden of age-related disorders while opening up new avenues for regenerative blood disease therapy. Numerous studies on the role of miRNAs in aging stem cells have revealed that changes in miRNA expression and their mRNA targets with age within a cellular environment play a critical role in cellular aging and the age-related phenotype. The progress in the comprehension of the miRNAs functions in aging might propose novel curative modalities. However, the role of miRNAs in senescence HSCs is still poorly understood. With the ongoing deepening of HSCs senescence investigation and the continuous progress of miRNAs as anti-aging techniques, the clinical usage of miRNAs in HSCs delaying human aging would gradually come to fruition.

## Data Availability

Not applicable.

## Abbreviations

HSCs: Hematopoietic stem cells
miRNAs: MicroRNAs
NSCs: Neural stem cells
MSCs: Mesenchymal stem cells
HFSCs: Hair follicle stem cells
ISCs: Intestinal stem cells
MuSCs: Muscle stem cells
BM: Bone marrow
NK: Natural killer cells
HSPCs: Hematopoietic stem-progenitor cells
EMPs: Erythro-myeloid progenitors
EPCs: Endothelial progenitor cells
ALDH: Aldehyde dehydrogenase
BMP: Bone morphogenetic protein
SDF1: Stromal cell-derived factor 1
ILs: Interleukins
Tpo: Thrombopoietin
Epo: Erythropoietin
MPPs: Multipotent progenitors
CD11B: Cluster of differentiation molecule 11B
bZIP: Leucine zipper
M-CSF: Macrophage Colony-Stimulating Factor
G-CSF: Granulocyte-CSF
TLRs: Toll-like receptors
PGE2: Prostaglandin E2
GDF11: Growth differentiation factor 11
mTOR: Mammalian target of rapamycin
AMPK: AMP-activated protein kinase
ROS: Reactive oxygen species
NAC: N-acetyl cysteine
ncRNA: Non-coding RNA
DNMT1: DNA methyltransferase 1
pol II: RNA polymerase II
RISC: RNA induced silencing complex
PBMC: Peripheral blood mononuclear cells
sp53: Super-p53
MEFs: Murine embryonic fibroblasts
CML: Chronic myelogenous leukemia
AML: Acute myeloid leukemia

## References

[1] Zhang Y (2022). MicroRNA-206 down-regulated human umbilical cord mesenchymal stem cells alleviate cognitive decline in D-galactose-induced aging mice. Cell death discovery.

[2] Jung Y, Brack AS (2014). Cellular mechanisms of somatic stem cell aging. Curr Top Dev Biol.

[3] Merlos-Suárez A (2011). The intestinal stem cell signature identifies colorectal cancer stem cells and predicts disease relapse. Cell Stem Cell.

[4] Tanimura S (2011). Hair follicle stem cells provide a functional niche for melanocyte stem cells. Cell Stem Cell.

[5] Yasamineh S (2022). Spotlight on therapeutic efficiency of mesenchymal stem cells in viral infections with a focus on COVID-19. Stem Cell Res Ther.

[6] Oveili E (2023). The potential use of mesenchymal stem cells-derived exosomes as microRNAs delivery systems in different diseases. Cell Communication and Signaling.

[7] Orkin SH, Zon LI (2008). Hematopoiesis: an evolving paradigm for stem cell biology. Cell.

[8] Rossi DJ (2005). Cell intrinsic alterations underlie hematopoietic stem cell aging. Proc Natl Acad Sci.

[9] Chivu-Economescu M, Rubach M (2017). Hematopoietic stem cells therapies. Curr Stem Cell Res Ther.

[10] Calvi LM, Link DC (2015). The hematopoietic stem cell niche in homeostasis and disease. Blood.

[11] Bunting KD, Qu CK (2014). The hematopoietic stem cell landscape. Methods Mol Biol.

[12] Mahla RS (2016). Stem cells applications in regenerative medicine and disease therapeutics. Int J Cell Biol..

[13] Challen GA (2010). Distinct hematopoietic stem cell subtypes are differentially regulated by TGF-β1. Cell Stem Cell.

[14] Beerman I (2010). Functionally distinct hematopoietic stem cells modulate hematopoietic lineage potential during aging by a mechanism of clonal expansion. Proc Natl Acad Sci.

[15] Dykstra B (2011). Clonal analysis reveals multiple functional defects of aged murine hematopoietic stem cells. J Exp Med.

[16] Yang L (2005). Identification of Lin–Sca1+ kit+ CD34+ Flt3–short-term hematopoietic stem cells capable of rapidly reconstituting and rescuing myeloablated transplant recipients. Blood.

[17] Liu YJ (2001). Dendritic cell subsets and lineages, and their functions in innate and adaptive immunity. Cell.

[18] Birbrair A, Frenette PS (2016). Niche heterogeneity in the bone marrow. Ann N Y Acad Sci.

[19] Rossi DJ, Jamieson CH, Weissman IL (2008). Stems cells and the pathways to aging and cancer. Cell.

[20] Klassert TE, Patel SA, Rameshwar P (2010). Tachykinins and neurokinin receptors in bone marrow functions: neural-hematopoietic link. J Receptor Ligand Channel Res.

[21] Ogawa T, Kitagawa M, Hirokawa K (2000). Age-related changes of human bone marrow: a histometric estimation of proliferative cells, apoptotic cells, T cells, B cells and macrophages. Mech Aging Dev.

[22] Harrison DE (1979). Mouse erythropoietic stem cell lines function normally 100 months: loss related to number of transplantations. Mech Aging Dev.

[23] Hao S, Chen C, Cheng T (2016). Cell cycle regulation of hematopoietic stem or progenitor cells. Int J Hematol.

[24] Koide S (2016). Setdb1 maintains hematopoietic stem and progenitor cells by restricting the ectopic activation of non-hematopoietic genes. Blood.

[25] Wang Z (2022). Loss of SIRT1 inhibits hematopoietic stem cell aging and age-dependent mixed phenotype acute leukemia. Commun Biol.

[26] Tajer P (2019). Ex vivo expansion of hematopoietic stem cells for therapeutic purposes: lessons from development and the niche. Cells.

[27] Souilhol C (2016). Developing HSCs become Notch independent by the end of maturation in the AGM region. Blood J Am Soc Hematol.

[28] Kenny PJ. Epigenetics, microRNA, and addiction. Dialogues Clin Neurosci. 2022. https://www.tandfonline.com/doi/full/10.31887/DCNS.2019.21.4/pkenny?scroll=top&needAccess=true&role=tab. 10.31887/DCNS.2014.16.3/pkenny PMC4214176 25364284

[29] Hu M (2021). MicroRNA-21 maintains hematopoietic stem cell homeostasis through sustaining the nuclear factor-B signaling pathway in mice. Haematologica.

[30] Georgantas RW (2007). CD34+ hematopoietic stem-progenitor cell microRNA expression and function: a circuit diagram of differentiation control. Proc Natl Acad Sci.

[31] Norouzi M (2019). Recent advances on nanomaterials-based fluorimetric approaches for microRNAs detection. Mater Sci Eng C.

[32] Hu J (2021). The potential use of microRNAs as a therapeutic strategy for SARS-CoV-2 infection. Adv Virol.

[33] Berindan-Neagoe I (2014). MicroRNAome genome: a treasure for cancer diagnosis and therapy. CA Cancer J Clin.

[34] Mohr AM, Mott JL (2015). Overview of microRNA biology. Semin Liver Dis.

[35] Lee Y (2004). MicroRNA genes are transcribed by RNA polymerase II. EMBO J.

[36] Foo JB (2021). Mesenchymal stem cell-derived exosomes and micrornas in cartilage regeneration: Biogenesis, efficacy, mirna enrichment and delivery. Pharmaceuticals.

[37] Yao S (2016). MicroRNA biogenesis and their functions in regulating stem cell potency and differentiation. Biol Proced Online.

[38] Di Leva G, Garofalo M, Croce CM (2014). MicroRNAs in cancer. Annu Rev Pathol.

[39] Morozova N (2012). Kinetic signatures of microRNA modes of action. RNA.

[40] Mokabber H, Vatankhah MA, Najafzadeh N. The regulatory role of microRNAs in the development, cyclic changes, and cell differentiation of the hair follicle. Process Biochem. 2022. https://rnajournal.cshlp.org/content/18/9/1635.short.

[41] Hou X-L (2021). DEAD-BOX RNA HELICASE 27 regulates microRNA biogenesis, zygote division, and stem cell homeostasis. Plant Cell.

[42] Dietrich C, et al. The emerging roles of microRNAs in stem cell aging. Exosomes Stem and Cells MicroRNA. 2018:11–26. https://link.springer.com/chapter/10.1007/978-3-319-74470-4_2. 10.1007/978-3-319-74470-4_2 29754172

[43] Potter ML (2021). MicroRNAs are critical regulators of senescence and aging in mesenchymal stem cells. Bone.

[44] Hammond SM, Sharpless NE (2008). HMGA2, microRNAs, and stem cell aging. Cell.

[45] So AY (2011). DNA methyltransferase controls stem cell aging by regulating BMI1 and EZH2 through microRNAs. PLoS ONE.

[46] Choi SW, Lee JY, Kang K-S (2017). miRNAs in stem cell aging and age-related disease. Mechanisms of Aging and Development.

[47] Conboy IM, Rando TA (2005). Aging, stem cells and tissue regeneration: lessons from muscle. Cell Cycle.

[48] Brunet A, Rando TA (2007). Aging: from stem to stern. Nature.

[49] Sharpless NE, DePinho RA (2007). How stem cells age and why this makes us grow old. Nat Rev Mol Cell Biol.

[50] Liu L, Rando TA (2011). Manifestations and mechanisms of stem cell aging. J Cell Biol.

[51] López-Otín C (2013). The hallmarks of aging. Cell.

[52] Signer RA, Morrison SJ (2013). Mechanisms that regulate stem cell aging and life span. Cell Stem Cell.

[53] Oh J, Lee YD, Wagers AJ (2014). Stem cell aging: mechanisms, regulators and therapeutic opportunities. Nat Med.

[54] Keyes BE, Fuchs E (2018). Stem cells: aging and transcriptional fingerprints. J Cell Biol.

[55] Vaziri H (1994). Evidence for a mitotic clock in human hematopoietic stem cells: loss of telomeric DNA with age. Proc Natl Acad Sci.

[56] Villeda SA (2014). Young blood reverses age-related impairments in cognitive function and synaptic plasticity in mice. Nat Med.

[57] Sinha M (2014). Restoring systemic GDF11 levels reverses age-related dysfunction in mouse skeletal muscle. Science.

[58] Baht GS (2015). Exposure to a youthful circulation rejuvenates bone repair through modulation of β-catenin. Nature Commun.

[59] Kowalczyk MS (2015). Single-cell RNA-seq reveals changes in cell cycle and differentiation programs upon aging of hematopoietic stem cells. Genome Res.

[60] Pang WW (2011). Human bone marrow hematopoietic stem cells are increased in frequency and myeloid-biased with age. Proc Natl Acad Sci.

[61] Yahata T (2011). Accumulation of oxidative DNA damage restricts the self-renewal capacity of human hematopoietic stem cells. Blood.

[62] Geiger H, Rudolph KL (2009). Aging in the lympho-hematopoietic stem cell compartment. Trends Immunol.

[63] Geiger H, De Haan G, Florian MC (2013). The aging haematopoietic stem cell compartment. Nat Rev Immunol.

[64] Henry CJ, et al. Declining lymphoid progenitor fitness promotes aging-associated leukemogenesis. Proc Natl Acad Sci. 2010:201005486. https://www.ncbi.nlm.nih.gov/pmc/articles/PMC3003039/#__ffn_sectitle. 10.1073/pnas.1005486107 PMC3003039 21098275

[65] Chambers SM (2007). Aging hematopoietic stem cells decline in function and exhibit epigenetic dysregulation. PLoS Biol.

[66] Dumble M (2007). The impact of altered p53 dosage on hematopoietic stem cell dynamics during aging. Blood.

[67] Miyamoto K (2007). Foxo3a is essential for maintenance of the hematopoietic stem cell pool. Cell Stem Cell.

[68] Florian MC (2012). Cdc42 activity regulates hematopoietic stem cell aging and rejuvenation. Cell Stem Cell.

[69] Beerman I (2013). Proliferation-dependent alterations of the DNA methylation landscape underlie hematopoietic stem cell aging. Cell Stem Cell.

[70] Michie KA, Löwe J (2006). Dynamic filaments of the bacterial cytoskeleton. Annu Rev Biochem.

[71] McKnight SL (2010). On getting there from here. Science.

[72] Grundling DA. Cloning and expression of human recombinant isoform a of Glycine-N-acyltransferase. Citeseer; 2012. https://citeseerx.ist.psu.edu/document?repid=rep1&type=pdf&doi=f15316fd7a300c89eb67477cde9859f3e2aef5df.

[73] Folmes CD (2012). Metabolic plasticity in stem cell homeostasis and differentiation. Cell Stem Cell.

[74] Jasper H, Jones DL (2010). Metabolic regulation of stem cell behavior and implications for aging. Cell Metab.

[75] Igarashi M, Guarente L (2016). mTORC1 and SIRT1 cooperate to foster expansion of gut adult stem cells during calorie restriction. Cell.

[76] Cerletti M (2012). Short-term calorie restriction enhances skeletal muscle stem cell function. Cell Stem Cell.

[77] Ito K, Suda T (2014). Metabolic requirements for the maintenance of self-renewing stem cells. Nat Rev Mol Cell Biol.

[78] Takubo K (2010). Regulation of the HIF-1α level is essential for hematopoietic stem cells. Cell Stem Cell.

[79] Beegle J (2015). Hypoxic preconditioning of mesenchymal stromal cells induces metabolic changes, enhances survival, and promotes cell retention in vivo. Stem cells.

[80] Tsai CC (2011). Hypoxia inhibits senescence and maintains mesenchymal stem cell properties through down-regulation of E2A–p21 by HIF-TWIST. Blood.

[81] Fang Y, Tang S, Li X (2019). Sirtuins in metabolic and epigenetic regulation of stem cells. Trends Endocrinol Metab.

[82] Rodriguez R, Fernandez A, Fraga M (2013). Role of sirtuins in stem cell differentiation. Genes Cancer.

[83] Mohrin M (2021). Mito–managing ROS & redox to reboot the immune system: tapping mitochondria & redox management to extend the reach of hematopoietic stem cell transplantation. Free Radical Biol Med.

[84] Mohrin M (2015). A mitochondrial UPR-mediated metabolic checkpoint regulates hematopoietic stem cell aging. Science.

[85] Amcheslavsky A, Jiang J, Ip YT (2009). Tissue damage-induced intestinal stem cell division in Drosophila. Cell Stem Cell.

[86] Biteau B (2010). Lifespan extension by preserving proliferative homeostasis in Drosophila. PLoS Genet.

[87] Tothova Z, Gilliland DG (2007). FoxO transcription factors and stem cell homeostasis: insights from the hematopoietic system. Cell Stem Cell.

[88] Tothova Z (2007). FoxOs are critical mediators of hematopoietic stem cell resistance to physiologic oxidative stress. Cell.

[89] Renault VM (2009). FoxO3 regulates neural stem cell homeostasis. Cell Stem Cell.

[90] McNeely T (2020). DNA damage in aging, the stem cell perspective. Hum Genet.

[91] Kenyon J, Gerson SL (2007). The role of DNA damage repair in aging of adult stem cells. Nucleic Acids Res.

[92] Borodkina A (2014). Interaction between ROS dependent DNA damage, mitochondria and p38 MAPK underlies senescence of human adult stem cells. Aging (Albany NY).

[93] Guachalla LM, Rudolph KL (2010). ROS induced DNA damage and checkpoint responses: influences on aging?. Cell Cycle.

[94] Sharma V (2016). Oxidative stress at low levels can induce clustered DNA lesions leading to NHEJ mediated mutations. Oncotarget.

[95] Rübe CE (2011). Accumulation of DNA damage in hematopoietic stem and progenitor cells during human aging. PLoS ONE.

[96] Ju Z (2007). Telomere dysfunction induces environmental alterations limiting hematopoietic stem cell function and engraftment. Nat Med.

[97] Rossi DJ (2007). Deficiencies in DNA damage repair limit the function of haematopoietic stem cells with age. Nature.

[98] Alter BP (2012). Telomere length is associated with disease severity and declines with age in dyskeratosis congenita. Haematologica.

[99] Moehrle BM, Geiger H (2016). Aging of hematopoietic stem cells: DNA damage and mutations?. Exp Hematol.

[100] Walter D (2015). Exit from dormancy provokes DNA-damage-induced attrition in haematopoietic stem cells. Nature.

[101] Yamamoto R (2013). Clonal analysis unveils self-renewing lineage-restricted progenitors generated directly from hematopoietic stem cells. Cell.

[102] Flach J (2014). Replication stress is a potent driver of functional decline in aging haematopoietic stem cells. Nature.

[103] Steensma DP (2015). Clonal hematopoiesis of indeterminate potential and its distinction from myelodysplastic syndromes. Blood.

[104] Aubert G, Lansdorp PM (2008). Telomeres and aging. Physiol Rev.

[105] Rudolph KL (1999). Longevity, stress response, and cancer in aging telomerase-deficient mice. Cell.

[106] Allsopp RC (2003). Effect of TERT over-expression on the long-term transplantation capacity of hematopoietic stem cells. Nat Med.

[107] Brunet A, Berger SL (2014). Epigenetics of aging and aging-related disease. J Gerontol Series A Biomed Sci Med Sci.

[108] Benayoun BA, Pollina EA, Brunet A (2015). Epigenetic regulation of aging: linking environmental inputs to genomic stability. Nat Rev Mol Cell Biol.

[109] Goldberg AD, Allis CD, Bernstein E (2007). Epigenetics: a landscape takes shape. Cell.

[110] Egger G (2004). Epigenetics in human disease and prospects for epigenetic therapy. Nature.

[111] Pal S, Tyler JK (2016). Epigenetics and aging. Sci Adv.

[112] Chen D, Kerr C (2019). The epigenetics of stem cell aging comes of age. Trends Cell Biol.

[113] Martin N (2013). Interplay between Homeobox proteins and Polycomb repressive complexes in p16INK4a regulation. EMBO J.

[114] Jacobs JJ (1999). The oncogene and Polycomb-group gene bmi-1 regulates cell proliferation and senescence through the ink4a locus. Nature.

[115] Trowbridge JJ (2009). DNA methyltransferase 1 is essential for and uniquely regulates hematopoietic stem and progenitor cells. Cell Stem Cell.

[116] Bröske A-M (2009). DNA methylation protects hematopoietic stem cell multipotency from myeloerythroid restriction. Nat Genet.

[117] Trowbridge JJ (2012). Haploinsufficiency of Dnmt1 impairs leukemia stem cell function through derepression of bivalent chromatin domains. Genes Dev.

[118] Sun D (2014). Epigenomic profiling of young and aged HSCs reveals concerted changes during aging that reinforce self-renewal. Cell Stem Cell.

[119] Rizo A (2008). Long-term maintenance of human hematopoietic stem/progenitor cells by expression of BMI1. Blood.

[120] Singh SK (2013). Sirt1 ablation promotes stress-induced loss of epigenetic and genomic hematopoietic stem and progenitor cell maintenance. J Exp Med.

[121] Liu L (2013). Chromatin modifications as determinants of muscle stem cell quiescence and chronological aging. Cell Rep.

[122] Okeke C (2022). HSC and miRNA Regulation with Implication for Foetal Haemoglobin Induction in Beta Haemoglobinopathies. Curr Stem Cell Res Ther.

[123] Luinenburg DG, de Haan G (2020). MicroRNAs in hematopoietic stem cell aging. Mech Aging Dev.

[124] Kurkewich JL (2018). The mirn23a and mirn23b microrna clusters are necessary for proper hematopoietic progenitor cell production and differentiation. Exp Hematol.

[125] Sandiford OA, et al. Human aging and cancer: role of miRNA in tumor microenvironment. Exosomes Stem Cells and MicroRNA. 2018:137–152. https://link.springer.com/chapter/10.1007/978-3-319-74470-4_9. 10.1007/978-3-319-74470-4_9 29754179

[126] Mehta A (2015). The microRNA-132 and microRNA-212 cluster regulates hematopoietic stem cell maintenance and survival with age by buffering FOXO3 expression. Immunity.

[127] Ooi AL (2010). MicroRNA-125b expands hematopoietic stem cells and enriches for the lymphoid-balanced and lymphoid-biased subsets. Proc Natl Acad Sci.

[128] Beerman I, Rossi DJ (2014). Epigenetic regulation of hematopoietic stem cell aging. Exp Cell Res.

[129] Yalcin S (2014). Microrna mediated regulation of hematopoietic stem cell aging. Blood.

[130] Herrera-Merchan A (2010). miR-33-mediated downregulation of p53 controls hematopoietic stem cell self-renewal. Cell Cycle.

[131] Oshima M, Iwama A (2014). Epigenetics of hematopoietic stem cell aging and disease. Int J Hematol.

[132] Darden DB (2020). Identification of unique mRNA and miRNA expression patterns in bone marrow hematopoietic stem and progenitor cells after trauma in older adults. Front Immunol.

[133] Grants JM (2020). Altered microRNA expression links IL6 and TNF-induced inflammaging with myeloid malignancy in humans and mice. Blood.

[134] Zhao JL (2013). MicroRNA-146a acts as a guardian of the quality and longevity of hematopoietic stem cells in mice. Elife.

[135] Nowicki M (2021). Alterations in microRNA expression during hematopoietic stem cell mobilization. Biology.

[136] Lechman ER (2016). miR-126 regulates distinct self-renewal outcomes in normal and malignant hematopoietic stem cells. Cancer Cell.

[137] Ping W (2018). mTOR signaling-related MicroRNAs and cancer involvement. J Cancer.

[138] Choi J (2016). MicroRNA-139-5p regulates proliferation of hematopoietic progenitors and is repressed during BCR-ABL–mediated leukemogenesis. Blood J Am Soc Hematol.

[139] Haetscher N (2015). STAT5-regulated microRNA-193b controls haematopoietic stem and progenitor cell expansion by modulating cytokine receptor signalling. Nat Commun.

[140] Zini R (2016). miR-382-5p controls hematopoietic stem cell differentiation through the downregulation of MXD1. Stem Cells Dev.

[141] Itkin T (2017). MicroRNA-155 promotes G-CSF-induced mobilization of murine hematopoietic stem and progenitor cells via propagation of CXCL12 signaling. Leukemia.

[142] Lam J (2018). miR-143/145 differentially regulate hematopoietic stem and progenitor activity through suppression of canonical TGFβ signaling. Nat Commun.

